# Novel biomarkers and therapeutic approaches for diabetic retinopathy and nephropathy: Recent progress and future perspectives

**DOI:** 10.3389/fendo.2022.1065856

**Published:** 2022-11-25

**Authors:** Ziyan Xie, Xinhua Xiao

**Affiliations:** Department of Endocrinology, Key Laboratory of Endocrinology, Ministry of Health, Peking Union Medical College Hospital, Chinese Academy of Medical Sciences & Peking Union Medical College, Beijing, China

**Keywords:** microvascular complications, diabetic retinopathy, diabetic nephropathy, biomarkers, therapy, artificial intelligence

## Abstract

The global burden due to microvascular complications in patients with diabetes mellitus persists and even increases alarmingly, the intervention and management are now encountering many difficulties and challenges. This paper reviews the recent advancement and progress in novel biomarkers, artificial intelligence technology, therapeutic agents and approaches of diabetic retinopathy and nephropathy, providing more insights into the management of microvascular complications.

## Introduction

Diabetes mellitus (DM) is a chronic metabolic disorder affecting more than 400 million people worldwide and is still on the rise. The long-standing hyperglycemia and genetic predisposition contribute to higher risks for macrovascular and microvascular complications among individuals with diabetes, which substantially places large financial and societal burden. Diabetic microvascular complications are associated with long term impairment and dysfunctions of various organs and systems including retina and kidney, which potentially result in blindness and end-stage kidney disorder, contributing significantly to the morbidity and mortality. These complications often already present in newly diagnosed diabetes and most patients may lost the opportunity to be diagnosed in the early stages to achieve clinically significant improvement. Therefore, early detection and novel treatment strategies are mandatory for alleviating progression and improving outcomes of microvascular complications. Nowadays, with the widely application of omics-technique, multiple novel biomarkers emerge as predictive and therapeutic targets for diabetic complications and increasing potential agents are in clinical trials or undergoing preclinical investigations. Furthermore, artificial intelligence (AI) is also developed and has been applicated in precision medicine, which facilitates the improvement of diagnosis and prognosis of microvascular complications. In this review, we highlighted the recent advances and new frontiers in the diagnosis and management of microvascular complications, especially focused on diabetic retinopathy (DR) and nephropathy (DN), providing perspectives on the clinical applications and implementation of novel biomarkers, diagnostic techniques and therapeutic agents.

## Diabetic retinopathy

### Novel biomarkers

DR remains the leading cause of visual impairment and blindness in working-age populations globally. Known risk factors including the duration of diabetes and poor glycemic control, however, cannot fully explain and predict the occurrence and progression of DR. And the present DR screening approaches all have different limitations such as poor accuracy, difficulty of obtaining high-quality images, invasive operation and high cost, which largely influence the early detection rate (1). Increasing evidence shows that pathological changes like inflammation and neuronal dysfunction may have occurred before retinal vasculature changes. Therefore, the development of a cost-effective biomarker that facilitates early risk assessment and accurate diagnosis is therefore urgently needed. The potential biomarkers of DR studied in recent years are listed in **Table 1**.

**Table 1:** Biomarkers for DR and DN.

Diabetic retinopathy				
Role	Profile	Biomarkers	Location	References
Inflammation	Cytokines	IL-1β, IL-2, IL-4, IL-5, IL-6, IL-8, IL-10, IL-17, MCP-1, TNF-α, IFN-γ	Serum, vitreous, aqueous	(2–7)
	Adhesion molecules	ICAM-1,VCAM-1	Serum	(2, 4)
	Immune cell markers	F4/80 mRNA, NF-κB, MIP-1α	Serum	(6, 8)
	Proteins	PTX3	Serum, aqueous	(9)
		RBP3	Vitreous, aqueous	(10, 11)
Angiogenesis	Growth factors	VEGF-A, VEGF-B, VEGF-C, VEGF-D, PlGF, FGF23	Serum, vitreous, aqueous	(12–14)
	Angiopoietin	ANGPTL3	Serum	(15)
Extracellular vesicles	Proteins	TNFAIP8, RANTES, CCR5	Plasma	(16–19)
Arteriosclerosis-associated parameters	Lipids	TG, LDL-C, sdLDL-C, apo	Serum	(14, 20, 21)
	Atherogenic index	(TC-(HDL-C))/HDL-C	Serum	(14)
	Atherogenic plasma index	(LDL-C/HDL-C)	Serum	(14)
Multi-omics	Metabolites	12-hydroxyeicosatetraenoic acid, 2-piperidone	Serum	(22)
	Proteins	lipophilin A, lactotransferrin, lysozyme C, lipocalin 1, mammaglobin B	Tears	(23)
	microRNAs	miR-9-3p, miR-431–5p, miR-200b-3p, miR-365-3p, miR-199a-3p, miR-146a-5p, miR-21, miR-34a, miR-145, miR-92a, miR-375	Plasma, serum, vitreous, aqueous	(24–29)
**Diabetic nephropathy**				
**Category**	**Profile**	**Biomarkers**	**Location**	**References**
Kidney injury	Proteins	NGAL	Urinary	(30–32)
		Cystatin C	Serum	(33)
		KIM-1	Urinary, plasma	(34)
		L-FABP	Urinary	(34)
		B2M	Plasma	(35)
	Angiopoietin	ANGPT1, ANGPT2	Urinary	(36, 37)
	Growth factors	VEGF, FGF, PDGF	Serum	(38, 39)
Inflammation	Chemokines	CCL19	Tubular sample	(40)
		CCL5	Urinary	(41)
		CCL15	Serum	(42)
	Cytokines	TNF-α, IL-1, IL-6, IFN-γ, MCP1	Urinary, serum	(34)
	Proteins	ICAM-1,VCAM-1, PAl-1, CRP, TNFR1, TNFR2	Serum	(34, 43)
Anti-inflammation	Proteins	Klotho	Serum	(44)
	Carotenoid	Lutein	Serum	(45)
Multi-omics	Proteins	CKD-273	Urinary	(46, 47)
	Metabolites	2-hydroxyisobutyrate,leucine, valine, pseudouridine, threonine, citrate	Urinary	(48)
	MicroRNA	miR192, miR-21, miR-29a-3p, miR-126-3p,miR-192-5p,miR-214-3p, miR-342-3p	Urinary, serum	(49)

#### Inflammatory biomarkers

Due to the indispensable role of inflammation in the pathogenesis of DR, close attention has been paid to inflammatory biomarkers for DR. A variety of clinical studies have provided evidence for the inflammatory biomarkers of DR. Multiple proinflammatory cytokines and adhesion molecules have been found increased in serum and ocular samples from both vitreous and aqueous humor of patients with DR, including interleukin family (IL-1β, IL-2, IL-4, IL-5, IL-6, IL-8, IL-10, IL-17), monocyte chemotactic protein-1 (MCP-1), tumor necrosis factor-α (TNF-α), interferon-γ (IFN-γ) and intercellular adhesion molecule-1 (ICAM-1), etc (2–5). And the level of IL-2, IL-5, IL-4, IL-6, IL-8, TNF-α, MCP-1 and macrophage inflammatory protein (MIP)-1α were significantly higher in early-onset proliferative diabetic retinopathy (PDR) patients compared to non-PDR (NPDR) and late-onset PDR, which facilitate evaluating the severity and predicting prognosis of DR in clinical practice (2, 6, 7). Besides, long pentraxin 3 (PTX3) has also been considered as a novel biomarker in DR. PTX3 is produced by endothelial cells in response to inflammation. Studies have shown elevated levels of PTX3 in the serum or aqueous humor of patients with DR compared with non-retinopathy or non-diabetic controls (9). Recently, King et al. recognized retinol-binding protein 3 (RBP3) as a potential biomarker for DR severity and progression. The level of RBP3 in aqueous humor was found to be reduced from mild NPDR, moderate and severe NPDR to PDR patients gradually (10). Elevated RBP3 level in the vitreous was correlated with lower levels of TNF-α, TNF-β and VEGF were associated with a lower risk of PDR (11).

Recent preclinical experiments also have gained progress in identification of potential biomarkers further used in DR detection and diagnosis. The main characteristics of inflammatory response in DR includes infiltration of immune cells such as macrophages, neutrophils, B and T cells, activation of microglia and enrichment of cytokines and chemokines (50, 51). Under normal condition, there is generally no immune cells within the vitreous body (52). However, in DR, which the blood-retinal barrier is disrupted, multiple immune cells (leukocytes and T, B cells) will enter the vitreous and trigger inflammatory reactions (53). Therefore, some immune cells and their related genes have been reported to be associated with DR, which may act as inflammatory biomarkers. Bioinformatics analysis identified 8 CD8+T lymphocytes-related genes linked to the occurrence and progress of diabetic macular edema (DME) in human macular samples and verified the expressions in DR mouse model (54). Similarly, hub genes of T-helper 17 (Th17) cells gained good diagnostic values in distinguishing PDR patients from normal subjects with the predictive role of the DR progression (NPDR and DME) (55). Furthermore, evidence have shown that inflammatory mediators initially rise in the retina and then enter the vitreous (12), implying the early predictive role of biomarkers in retina. Several monocyte/macrophage markers (e.g.,F4/80mRNA, CCL2) and glial cells markers (e.g.,NF-κB, IL-17) in retina are found correlated with the progression of DR in rat models (8), which need further confirmation in clinical practice.

#### Angiogenesis biomarkers

Angiogenesis is an critical pathological factor in the occurrence and progression of DR. Vascular endothelial growth factor (VEGF) is the main angiogenic regulator which serves as a promising predictive biomarker and target for the treatment of DR. Accumulating evidence has suggested the circulating or even tear’s level of VEGF, significantly increased in PDR compared with NPDR, which predict DR severity (13). Clinical studies indicate that serum VEGF-A, VEGF-C, VEGF-D and placental growth factor (PlGF), and vitreous and aqueous VEGF-A and PlGF are positively correlated with the severity of DR and are strong predictors for DR occurrence in diabetic individuals (12, 14). And anti-VEGF injection in PDR patients significantly reduced the levels of VEGF-A in aqueous, which suggests the potential role of aqueous VEGF in evaluating the efficacy of PDR therapies (12). Besides, circulating angiopoietin-like 3 (ANGPTL3) is also considered as a promising biomarker which independently and strongly associated with DR onset and development (15). Moreover, recent studies revealed more candidate angiogenic factors like ITGA7, FGF23, THBS1, COL1A1, MAPK13, and AIF1 in early stage of DR (56), which may be novel intervention targets. Exploring the mechanisms of angiogenesis also could facilitate early recognition of DR.

#### Extracellular vesicles

In recent years, extracellular vesicles (EVs) have gained increasingly attention as potential sources of new biomarkers for multiple diseases. According to their size and biogenesis, EVs can be classified as exosomes, microvesicles, or apoptotic bodies. It was found that hyperglycemia leads to the increase of EVs from donor cell. The molecular profiles and origins of EVs are distinct between DR and normal, presenting proinflammatory and proangiogenic properties. 90 proteins in the proteomic profiles of plasma EVs significantly changed between DR and non-DR subjects, among them, tumor necrosis factor-α-induced protein 8 (TNFAIP8) was increased in DR patients (16). *In vitro* experiment confirmed the angiogenic role of TNFAIP8 in retinal microvascular endothelial cell, indicating that TNFAIP8 in plasma EVs may act as a new biomarker for DR (16). Aleksandra et al. have described that the plasma level of microvesicles containing RANTES and CCR5 receptors, which both act as inflammatory and proangiogenic factors, were significantly higher in NPDR than diabetic patients without DR, and were positively associated with the progression of DR (17). Furthermore, Ogata et al. have reported gradually elevated levels of platelet- and monocyte-derived EVs in the early and advanced stage of DR (18, 19). These platelet-derived EVs induce oxidative stress and monocyte-derived EVs exaggerate inflammatory responses, leading to retinal vascular damages during the development of DR. Therefore, circulating EVs as carriers of proteins and RNAs, are not only the messengers for cell-cell communications, but serve as important biomarkers for prediction of disease occurrence and progression.

#### Arteriosclerosis-associated biomarkers

Plasma lipid parameters not only predict cardiovascular outcomes but also act as markers for microvascular complications. Serum total cholesterol (TC) and low-density lipoprotein cholesterol (LDL-C) are found significantly elevated in DR patients, acting as risk factors for the presence of DR. Besides, the atherogenic plasma index calculated as (LDL-C/HDL-C) and atherogenic index calculated as (TC-(HDL-C))/HDL-C also possess great values in predicting the onset and severity of DR compared with the traditional lipid indexes (14). Apolipoprotein (apo) profiles in non-DR, NPDR and PDR subjects revealed a panel of apos as independent risk factors for the occurrence and severity of DR and two apos as protective factors, which could be further used as biomarkers for predicting DR (20). Additionally, some lipid parameter has also been suggested to guide treatment of DR. For example, small dense low-density lipoprotein cholesterol (sdLDL-C) was identified as a sensitive biomarker for evaluating the need of laser treatment for DR patients (21).

#### Multi-omics related biomarkers

Development of high-throughput sequencing techniques allows the detection and quantification of the overall and dynamic changes in genome, transcriptome, proteome and metabolome, which produce a large amount of date in a short time and provide insight in identification of novel biomarkers (57). A recent metabolomics study observed a serum metabolites panel associated with DR occurrence and development. Serum 12-hydroxyeicosatetraenoic acid and 2-piperidone exhibited better diagnostic value than hemoglobin A1c (HbA1c) in differentiating DR from non-DR diabetes, which can be used for detection of early-stage DR (22). Besides, proteomics analysis from tears of DR patients uncovered multiple proteins changed correlating with the occurrence and severity of DR, providing evidence and targets for tear-based protein biomarkers for early diagnosis of DR (23). Furthermore, non-coding RNAs, especially microRNAs, are also the most well-studied biomarkers of DR (8). MicroRNAs have been shown to regulate multiple pathological processes during DR, including cell proliferation, apoptosis, inflammation and microcirculation impairments and exerted differential expressions in blood and vitreous samples from DR patients (24, 58). Therefore, microRNAs may act as promising biomarkers for early diagnosis and progression of DR.

### Artificial intelligence

Diagnosis and monitoring the progression of DR heavily reliance on imaging, artificial intelligence (AI) has been a pioneer in the early detection and screening of DR. Based on the rich data generated by imaging techniques, using machine learning and deep learning, AI is now being applied in facilitating the early recognition, prognosis and treatment selection. Pivotal studies have demonstrated the clinical benefits of AI system for detection and screening of DR. A prospective multicenter study evaluated the efficacy of the AI DR detection system (EyeArt) in detecting more-than-mild DR and vision-threatening DR (59). The findings found high sensitivity and specificity of the AI system in detecting more-than-mild DR (95.5% and 85%, respectively) and even vision-threatening DR (95.1% and 89%), without physician assistance. Dai et al. (60) developed DeepDR system based on deep learning method, which can automatically detect the whole course of DR from mild to PDR, providing real-time feedback to the quality of fundus images as well as the recognition and segmentation of fundus diseases. Furthermore, AI techniques have also been used in DR prognosis and treatment efficacy judgement. A prospective study evaluated the potential of AI using optical coherence tomography data including segmentation of intraretinal cystoid fluid (IRC), retinal layer segmentation and subretinal fluid, to predict the prognosis of patients with DME. The findings showed that IRC possessed the greatest predictive value for best-corrected visual acuity (BCVA) at baseline, while IRC and total retinal thickness had greater prognostic value for BCVA after 4 and 12 weeks. The application of AI transforms descriptive data into information and factors that can be used for prediction. The establishment of predictive models for the prognosis will encourage patients to pursue aggressive treatment and make optimal treatment options (61). Early detection is critical for DR management and reducing the blindness rate of DR. However, current DR screening faces the dilemma of insufficient ophthalmologists for standardized image reading and lack of awareness for DR screening. AI with the characteristics of high accuracy, easy to copy and promote, is promising to make up for staff shortages and facilitate early screening, intelligent fundus reading and intelligent diagnosis of DR in clinical practice. Until now, both the EyeArt and DeepDR systems have been approved for clinical practice. Similarly, other AI systems like IDx-DR (62), RetmarkerDR (63), Singapore SERI-NUS (64) and so on, also obtain good results in DR screening in different populations.

### Novel therapeutic approaches

Treatment for DR generally includes systemic control, laser photocoagulation, and pharmacotherapies targeting mediators involved in pathogenesis of DR. The recent progress of therapeutic research in DR continues the previous academic concerns including anti-VEGF therapy, anti-inflammatory treatment, traditional Chinese medicine, and precision drug delivery, etc.

#### Anti-VEGF therapy

Approved in 2013 by US FDA, Anti-VEGF drugs are currently the first line therapy of DME with vision loss. Several studies, such as RESTORE (65), VISTA and VIVID (66) study have shown that the emergence of anti-VEGF therapy led to significant clinical improvements compared to laser therapy alone. Besides, a recent randomized clinical trial observed that combination of intravitreal bevacizumab injections and laser photocoagulation was more effective in preventing neovascularization and ameliorating visual field than pan-retinal photocoagulation (PRP) or bevacizumab alone in PDR patients (67). The combined protocol reduced the adverse effects of full PRP and need fewer injections and visits, suggesting that for some high-risk patients, anti-VEGF combined with PRP therapy may be more helpful in delaying the progression and improving the adherence of injections in DR. Furthermore, PANORAMA study found that anti-VEGF therapy also improve NPDR without DME (68). Compared with sham, intravitreal aflibercept significantly improved retinopathy severity scale and reduced vision-threatening adverse effects in moderately severe to severe NPDR. Though anti-VEGF therapy have gained benefit in clinical trials, frequent injections, close monitoring and heterogeneous response of patients are current barriers to achieve optimal outcomes in real world. Recently, long-acting and slow-release anti-VEGF agents have shown benefits in clinical trials. KESTREL and KITE study demonstrated that brolucizumab, a new agent targeting VEGF-A and facilitating high and sustained molar concentration, improved BCVA and reduced central subfield thickness (CSFT), subretinal and/or intraretinal fluid in DME patients during 52 weeks therapy (69). Additionally, Abicipar pegol, a VEGF-A inhibitor with longer half-life and higher affinity than ranibizumab, has also shown functional and anatomical improvements with fewer injections compared with ranibizumab administered every 4 weeks over a period of 28 weeks in DME patients (70). Alternatively, the port delivery system for anti-VEGF drugs also allows long-term, continuous delivery of ranibizumab into the vitreous, which maintains optimal vision and anatomic outcomes with reduced number of injections and gains high treatment satisfaction from patients (71, 72). This technology is now applied in patients with neovascular age-related macular degeneration in clinical trial stage and is expected to be further used in the treatment of DME. New agents beyond the VEGF pathway are also developed to optimize the efficacy and compliance of DR treatment. Faricimab is a novel antibody inhibiting angiopoietin2 (ANGPT2) and VEGF-A with high affinity and specificity. Two phase III trials indicated that faricimab administration achieved vision gains and improved anatomical outcomes with fewer visits and increased dosing compared with aflibercept in DME treatment (73). Furthermore, gene therapy is of great interest in the treatment of DR. RGX-314 uses the NAV AAV8 vector to deliver anti-VEGF monoclonal antibody fragment and is promising to maintain continuous expression of anti-VEGF-A protein in retina with a single administration (74). And an ongoing phase II clinical trial (ALTITUDE) is now conducted to evaluate the safety, tolerance and efficacy of RGX-314 in patients with moderate to severe NPDR and mild PDR. Further research is still needed to determine the safety, efficacy, durability and targeted population of gene therapy prior to implementing into clinical practice. Anti-VEGF agents targeting DR are summarized in **Table 2**.

**Table 2:** Anti-VEGF agents for treatment of DR.

Agents	Mechanism	Study type	Status	Regimen	Outcomes
Ranibizumab (65)	Anti-VEGF	RCT Phase III	Completed	Intravitreal 0.5 mg	Superior visual acuity and central macular thickness improvement in DME compared with laser
Bevacizumab (75)	Anti-VEGF	RCT	Completed	Intravitreal 1.25mg	Significant central macular thickness reduction and visual acuity improvement in DME compared to laser
Aflibercept (66, 68)	Anti-VEGF	RCT Phase II & III	Completed	Intravitreal 2mg	Superior in improving visual acuity vs.laser in DME and PDR; Significantly improving retinopathy severity scale and reducing adverse effects in NPDR
Brolucizumab (69)	Anti-VEGF	RCT Phase III	Completed	Intravitreal 3 mg/6 mg	6 mg showed superior improvements in central subfield thickness and lower adverse effects vs aflibercept
Abicipar pegol (70)	Anti-VEGF	RCT Phase II	Completed	Intravitreal 2 mg	Functional and anatomical improvements with fewer injections compared with ranibizumab in DME
Faricimab (73)	VEGF and ANGPT2 inhibitor	RCT Phase III	Completed	Intravitreal 6 mg	Superior vision gains and anatomical outcomes improvements with fewer visits compared with aflibercept in DME
Conbercept (76)	Anti-VEGF	Retrospective	Completed	Intravitreal	Significant visual acuity improvement compared to baseline in DME Non-inferior to ranibizumab
Pegaptanib (77)	Anti-VEGF	RCT Phase II & III	Completed	Intravitreal 0.3 mg	Superior visual acuity improvement vs. sham in DME
RGX-314	Anti-VEGF (gene therapy)	RCT Phase II	Ongoing	Intravitreal 2.5x10^11^ ~5x10^11^genomic copies per eye	Phase II trial ongoing

#### Anti-inflammatory therapy

Inflammation plays a critical role in the pathogenesis of DR, and anti-inflammatory agents have shown functional and anatomical improvement in DR and DME. Clinical studies demonstrated that the visual outcomes of intravitreal dexamethasone injection were comparable with the anti-VEGF group after 1 year (78). DR patients who did not respond to anti-VEGF drugs, switching to dexamethasone sustained-release therapy may still reduce macular edema and improve vision (79). *In vitro* experiments implied that dexamethasone reduced the level of cytokines, including IL-1β, IL-6, and TNF-α in retinal ganglion cell and Müller cells, which alleviated hyperglycemia induced inflammation and improved cell survival rate (80). In addition to glucocorticoids, key inflammatory factors can be used as new targets for DR treatment. CD40 is a critical driver of DR which induces proinflammatory cytokines release in myeloid cells, and treatment blocking CD40 signaling decreased the level of inflammatory molecules in retina of diabetic mice, providing evidence for the potential of CD40 in DR treatment (81). Moreover, nuclear factor of activated T cells (NFAT) and RBP3 are also important biomarkers involved in retinal inflammation. Inhibition of NFAT prevented retinal vascular leakage and inflammation in DR mice model; while intravitreal injection of recombinant human RBP3 reversed high glucose induced retinal vascular dysfunction and inflammatory cytokine elevations in STZ rats. The role of these inflammatory biomarkers in the progression of DR needs further research, and they are expected to become novel targets in pharmacotherapeutics.

#### Renin-angiotensin-aldosterone system therapy

RAAS activation potentially has a role in the development of end-organ damage, and has been considered as a risk factor for DR. Activation of the angiotensin receptor 1 (AT1-R) induces the progression of DR by stimulating multiple pathways involved in the pathogenesis including advanced glycosylation end-products (AGE) accumulation, inflammation, oxidative stress, and several crucial mediators of angiogenesis such as VEGF (82). Clinical and animal experiments showed that the RAAS is activated in diabetic retinopathy and its inhibitors may exert protective role against retinal damage (83, 84). Several clinical trials indicated that ACE inhibitors and/or ARBs treatments reduced the incidence of DR in hypertensive diabetic people (85–87). However, there also exists studies that showed no benefit in DR incidence or DR progression from the ACE inhibitor and ARB therapy (85, 88), which indicates that mechanism other than RAAS also responsible for the progression of DR, and further research working on the efficacy of RAAS inhibitor to prevent the progression of DR in diabetic people are still needed.

#### Nanotechnology

Recently, nanotechnology has been widely applied in the medical field, broaden the horizon of new drug discovery, drug delivery and precision treatment. The diameter of nanomolecules ranges from 1 to 100 nanometers, enabling the drugs pass through the blood-retinal barrier. Nanoparticle-based delivery of triamcinolone acetonide are safe and long-lasting and significantly improved anatomic outcome and functional activity of retina in DR rat model (89). In addition, multiple micro RNAs (miRNA) and DNAs can also be delivered by nanomaterials, which are expected to delay DR progress and promote retinal regeneration.

## Diabetic nephropathy

### Novel biomarkers

Early recognition of DN is key to preventing renal function reduction, however, biomarkers currently used in clinical practice such as albumin, creatinine and eGFR do not sufficiently predict and assess the progression of DN (90). Therefore, novel biomarkers to predict the risk of functional decline have been urgently sought. In recent years, multiple efforts have been made to identify new reliable and sensitive biomarkers for the early diagnosis and monitoring of DN (**Table 1**).

#### Kidney injury biomarkers

Several biomarkers indicating kidney injury are being developed (34). Neutrophil gelatinase-associated lipocalin (NGAL) is a well-studied tubular damage marker. DN patients with normo-albuminuria have present increased levels of urinary NGAL, which implies that tubular damage may occur in very early stages (30–32). Besides, kidney injury molecule 1 (KIM-1) and β-2-Microglobulin (B2M) have also been extensively studied in DN. Longitudinal studies indicated that high level of KIM-1 had a strong correlation with higher risk of eGFR decline and increased risk of DKD (43, 91). Evidence also showed that diabetic individuals with elevated B2M levels had a higher risk for DN, which may serve as promising predictors of DN progression in diabetic patients (35). ANGPT are vascular growth factors that promote angiogenesis and vascular repair and play a crucial role in the glomerular capillaries (36). Elevated level of urinary ANGPT2 was found in T2D patients with renal damage and was associated with albuminuria (36). Inversely, ANGPT1 has exerted a protective effect against renal function decline and reduced level of ANGPT1 was detected in early diabetic kidney disease (37). Other growth factors such as VEGF and fibroblast growth factor (FGF) have also been previously evaluated as important biomarkers and targets in DN diagnosis and progression (38, 39). Furthermore, recent studies illustrated that serum cystatin C, another marker of tubular damage, shows better predicting value of eGFR decline in diabetes patients than creatinine (33).

#### Inflammatory/anti-inflammatory biomarkers

Markers involved in inflammation are also highly reported (34). Chemokines such as CCL19,CCL5 and CCL15 have been identified as critical genes of DN. Bioinformatics analysis and *in vitro* experiments revealed that CCL19 was significantly upregulated in tubular samples of DN patients (40). Besides, urinary level of CCL5 was correlated with renal function reducing and the extent of renal interstitial fibrosis (41). While serum CCL15 is found negatively correlated with eGFR and independently associated with high DN risk in T2D patients (42). In addition, prospective studies reported that diabetic patients with higher plasma levels of TNFR1, TNFR2 and MCP1 had increased risk of progression of DN (43). On the other hand, some anti-inflammatory markers have also been studied in DN. Klotho protein mainly expressed in the kidney and can suppress the inflammatory response. A recent meta-analysis found that the soluble Klotho was significantly lower in DN than that in control, and this decrease can be detected in the early stages of DN (44). Lutein is an oxygenated carotenoid with antioxidation and anti-inflammatory effects. Serum lutein level is negatively associated with the risk of DN and possesses a good diagnostic value of DN with an AUC of 0.779 (45).

#### Multi-omics related biomarkers

The urinary proteomic CKD273 score was used to quantify the risk for the new onset of albuminuria. CKD-273 was validated in T2D cohorts and shown to predict the progression of albuminuria in DN (46, 47). Besides, metabolomics of urine samples from 2670 T1D individuals revealed five urinary metabolites closely associated with kidney disease progressing, and the level of 2-hydroxyisobutyrate reflected the progression of DN in individuals with normo-albuminuria (48). Urinary or serum miRNAs also emerge as new biomarkers for DN depending on the microarray and RNA-sequencing techniques (49).

### Novel therapeutic approaches

The current therapy for DN including glycemic control, blood pressure and cholesterol management, focus on the systematic control of DR. As the emergence of abundant targets involved in DN, the direction of DN treatment investigation has been progressed to focus on molecular mechanism and target the critical molecules or signaling pathways in the progression of DN.

#### Anti-inflammatory agents

Pentoxifylline (PTF) is a methylxanthine derivate and plays an anti-inflammatory role in kidney disorder progression. An early meta-analysis summarized that PTF may reduce proteinuria in patients with DN (92). The PREDIAN Trial and a recent randomized clinical trial both confirmed that the addition of PTF to renin-angiotensin system antagonists resulted in a more significant reduction of urine albumin excretion and slowed the progression of renal function decline (93, 94). Besides, some chemokine antagonists also exert beneficial effects in clinical studies. CCX140-B, a selective MCP-1 inhibitor, significantly reduced albuminuria by 18% in DN patients (95). A phase II study in T2D patients with albuminuria demonstrated that emapticap pegol (NOX-E36), another antagonist of MCP-1, was safe and well tolerated during administration, and significantly improved urinary albumin/creatinine ratio (ACR) compared to the placebo group (96). Klotho could inhibit the expression of MCP-1 and ICAM-1, resulting in lower accumulation of macrophages, thus exerting anti-inflammatory function and reduced tubulointerstitial injury (97).

#### Anti-fibrotic agents

Some agents have been found to target renal cell function for anti-fibrotic effect in DN. Oxymatrine prevented renal extracellular matrix deposition by inhibiting the epithelial-to-mesenchymal transition (EMT) process and ultimately attenuated tubulointerstitial fibrosis (98). The extract of P. fallax has been demonstrated to downregulate the expression of ECM proteins, such as FN, Col IV, MMP-9, and MMP-2, therefore protecting glomerular mesangial cell from high glucose induced fibrosis (99).

#### Renin-angiotensin-aldosterone system therapy

The RAAS plays an important role in renal disease. In DN, the RAAS has been linked with changes in intraglomerular hemodynamics as well as structural alterations in both the glomerulus and tubulointerstitium (100). Growing evidence has revealed that RAAS inhibitors blocked the development of kidney diseases, manifesting as improved proteinuria and well-maintained renal function (101). It has been shown that ACEI or ARB which block the RAAS delayed the development of DN and reduced the incidence of end-stage renal disease in DN patients with large albuminuria (102).

#### Novel anti-diabetic drugs

Recently, the emergence of novel anti-diabetic drugs not only improve the management of hyperglycemia, but also obtain heart and kidney benefits. SGLT2 inhibitors are oral hypoglycemic drugs and exhibit renoprotective effects. Studies demonstrated that SGLT2 inhibitors therapy decreased albuminuria, prevented GFR decline, and reduced need for renal replacement therapy or death from kidney causes in diabetes patients (103, 104). Besides, data suggested DPP-4 inhibitors and GLP-1 agonists also have renoprotective effect in diabetic patients through antioxidant, anti-inflammatory and antifibrotic mechanisms, which prevent DN occurrence and development (105, 106).

#### Endothelin receptor blocker

Endothelin (ET) is a kind of vasoconstrictor which has vasoactive, inflammatory, and profibrogenic characters and is significantly correlated with kidney disorders. Animal experiments suggested that ET receptor antagonists reduced proteinuria and exerted nephroprotective effect in experimental models of DN (107). Phase III clinical trials also showed that ET receptor antagonists decreased albuminuria in patients with DN (108). However, the safety and side effects (congestive heart failure) of ET receptor blockers require further investigation.

#### Vitamin D supplementation

Some clinical trials and observational studies have supported the benefits of vitamin D supplementation in DN treatment. A randomized control study revealed that paricalcitol decreased urinary albumin excretion rate by 18% in T1D patients compared to placebo (109). Similarly, an observational study also showed that oral cholecalciferol therapy for 4 months significantly reduced the albuminuria in T2D patients (110). A meta-analysis including 20 randomized clinical trials with total 1464 DN patients suggested that vitamin D supplement (calcitriol, alfacalcidol and vitamin D3) improved 24-hour urine protein and urine albumin excretion rate, as well as inflammatory indexes, such as hs-CRP, TNF-α and IL-6 (111). But there was no significant difference in serum creatine, eGFR or HbA1c. Therefore, more trials are needed to confirm the therapeutic value of vitamin D supplementation in DN for reaching a consensus and recommendation.

#### Nanotechnology

Recent research assembled synthetic high-density lipoprotein (sHDL) with a liver X receptor (LXR) agonist, which aim to deliver LXR agonists to kidney and promote the removal of excessive lipids from mesangial cells, thus attenuating inflammation and recovering renal function, and the efficacy of sHDL nanoparticle has been confirmed in DN animal experiments (112).

## Link between DR and DN

DR and DN are both major microvascular complications of diabetes. DR is the leading cause of blindness in adults aged 20-74 years, with almost all type 1 diabetes and 60% of type 2 diabetes occurring after 20 years. DN is one of the major causes of end stage renal disease (ESRD), contributing to significant morbidity and cardiovascular mortality (113). The connection between DR and DN can be addressed in many aspects. Firstly, these two complications share common pathogenetic mechanisms (114). The main biological mechanisms of microvascular complications include advanced glycation end products (AGEs) accumulation, polyol pathway activation, oxidative stress, inflammatory responses (e.g., activation of NF-kB, adipokines, chemokines, adhesion molecules and proinflammatory cytokines), hemodynamic alterations (e.g., RAAS activation) and growth factors overexpression (e.g., VEGF is one of the most important factors in the progression of DR and DN). Secondly, the similar underlying pathogenetic mechanisms contribute to the overlap of biomarkers for predicting the progress of DR and DN. As we discussed previously, DR and DN share many common biomarkers, such as inflammatory biomarkers, angiogenetic biomarkers, and lipid profiles. Therefore, combination of these biomarkers could facilitate detecting early disease affecting both systems, and therapeutic strategies targeting their common markers may also gain retinal and renal benefits. Moreover, DR and DN have a predictive effect on each other. Accumulating evidence suggested that the presence of DR increased the incidence of DN in diabetic patients and DR has been considered as the predictor of DN, especially PDR as a more sensitive predictor (115, 116). Yang et al. (117) found that haptoglobin, a urine proteome specific for PDR could serve as an indicator complementing to urine albumin to predict renal dysfunction in patients with T2D. Likewise, DN can also predict DR to some extent. A prospective ten-year follow-up study demonstrated that eGFR and ratio of urine albumin to creatinine (ACR) served as sensitive biomarkers to predict the incidence of DR (118). And serum creatinine and decreased eGFR has been shown to be associated with the progression and severity of DR in diabetic patients (119, 120). Although the onset of retinopathy symptoms is very hidden, with the development of fundus examination and AI, accurate and early recognition of DR gains clinical feasibility. However, the early stage of DN lacks specific clinical symptoms and requires kidney biopsy to confirm the diagnosis, which is the greatest challenge for clinical promotion. Microalbuminuria, the most used indicator, however, its accuracy remains controversial. Therefore, considering the close link between DR and DN, it is promising to replace invasive and unpredictable detection of other microvascular complications with noninvasive, simple, and low-cost ophthalmoscopy. And vice versa, it also useful to screen patients with kidney disorders for associated retinal diseases. Comprehensive clinical evaluation in patients with CKD or ESRD should include external ophthalmoscopy and direct ophthalmoscopy. These recommendations may facilitate early recognition of both retinal and renal damages in diabetic patients and provide evidence for multi-factorial approach and potential multi-target therapeutic strategies.

## Perspectives

In conclusion, sensitive and cost-effective biomarkers facilitating early identification guarantees optimal treatment of DM related microvascular complications. Multi-omics techniques provide huge data for candidate biomarker discovery, however, most of them remains further confirmation in clinical practice. The application of AI technology may also be expected to achieve effective diagnosis, efficacy determination, and prognosis of microvascular complications. Furthermore, in view of the close association between DR and DN, biomarkers and therapeutic strategies targeting their common pathophysiology mechanism such as VEGF and inflammation are key research directions in the future. Novel agents targeting multifactor which will complement current therapies in effects, such as SGLT-2 inhibitor and DPP-4 inhibitor, etc., are expected to benefit more patients. Finally, nanotechnology have shown incomparable benefits in non-invasive and precise drugs delivery. The application of nanomaterial-based drug delivery systems in microvascular complications therapy is of great potential and interests.

## Author contributions

Manuscript drafted by ZX, edited and revised by XX. All authors contributed to the article and approved the submitted version.

## Fundings

This work was supported by the grants from the National Natural Science Foundation of China (No. 82170854, 81870579, 81870545, 81570715, 81170736) and National High Level Hospital Clinical Research Funding (2022-PUMCH-C-019).

## Conflict of interest

The authors declare that the research was conducted in the absence of any commercial or financial relationships that could be construed as a potential conflict of interest.

## Publisher’s note

All claims expressed in this article are solely those of the authors and do not necessarily represent those of their affiliated organizations, or those of the publisher, the editors and the reviewers. Any product that may be evaluated in this article, or claim that may be made by its manufacturer, is not guaranteed or endorsed by the publisher.
